# IQGAP1 participates in endothelial cell apoptosis and regulates atherosclerosis by targeting YAP

**DOI:** 10.1371/journal.pone.0328345

**Published:** 2025-07-14

**Authors:** Shaojun Huang, Yao Cheng, Chengxin Zhang

**Affiliations:** 1 Panzhihua Central Hospital, Panzhihua, Sichuan Province, China; 2 Department of Cardiovascular Surgery, The First Affiliated Hospital of Anhui Medical University, Hefei, Anhui Province, China; Tabriz University of Medical Sciences, IRAN, ISLAMIC REPUBLIC OF

## Abstract

Endothelial cell (EC) apoptosis plays a crucial role in the onset and progression of atherosclerosis (AS). IQGAP1 is highly expressed in various tissues and affects cell growth, development and death. However, the complete elucidation of the influence of IQGAP1 on EC apoptosis and AS remains unclear. This study endeavored to establish that IQGAP1 expression is augmented in the aortic wall of AS mice. Additionally, the present study demonstrated increased BAX and cleaved caspase-3 expression along with notably reduced BCL-2 expression within the aortic wall of mice with AS. After transfecting small interfering RNA of IQGAP1 (Si-IQGAP1) into normal or palmitic acid (PA)-treated human umbilical vein endothelial cells (HUVECs) cultured in vitro, the apoptotic rate decreased. Furthermore, western blot analysis demonstrated reduced expression pro-apoptotic proteins, namely BAX and cleaved caspase-3, accompanied by increased expression of the anti-apoptotic protein BCL-2. Simultaneously, the key protein of the Hippo signaling pathway, YAP, showed increased expression, whereas phosphorylated YAP expression decreased. However, subsequent to the overexpression of IQGAP1, the trajectory of the aforementioned parameters was reversed. In addition, the knockdown of YAP promoted the apoptosis rate in co-transfected Si-IQGAP1 and Si-YAP group. In conclusion, IQGAP1 potentially facilitates apoptosis in HUVECs, thereby contributing to AS initiation and progression. This mechanism may be partly attributed to the modulation of pivotal proteins, including YAP.

## 1. Introduction

Currently, atherosclerotic cardiovascular disease (ASCVD) has the highest global incidence and mortality rate, resulting in numerous fatalities annually [1]. Contemporary investigations have proposed that atherosclerosis (AS) is the foremost pathological basis of ASCVD; however, the potential pathogenic mechanisms of AS remain largely undefined. The increased apoptosis of endothelial cells (ECs) induced by lipid deposition plays a pivotal role as the foremost initiating factor of AS [2]. Lipid deposition can regulate the expression of BAX, cleaved caspase-3, and other apoptosis-related proteins by regulating the NF-kB signaling pathway and transcription factors, which causes and further advances the development of apoptosis and AS [3]. Lipid deposition promotes EC apoptosis through ROS-dependent JNK and p38 MAPK signaling, resulting in AS [4].

IQ motif containing GTPase activating protein 1 (IQGAP1), a 190-KDa protein, can serve as a scaffold protein and bind hundreds of proteins, such as Cdc42, Rac1 [5] and ERK1 [6], to participate in cellular adhesion, migration, and polarization [7]. Recently, the role of IQGAP1 in the regulation of apoptosis has attracted significant attention. Studies have suggested that IQGAP1 promotes the apoptosis of glomerular podocytes in the human cancer cell line HT1080 and osteoprogenitor MC3T3-E1 cells [8–10]. Some studies have shown that IQGAP1 can act as a proto-oncogene to promote tumor cell proliferation, migration, and invasion [11]. However, the regulatory mechanism between IQGAP1 and endothelial cell apoptosis in atherosclerosis is not yet clear. Recent evidence has revealed that IQGAP1 participates in nuclear functions and modulates transcription. IQGAP1 directly interacts with Nrf2 [12] to influence its function and enhance its stability, which is similar to its effect on β-catenin [13]. The role of IQGAP1 as a proto-oncogene in inflammation and ECs has received increasing attention. IQGAP1 promotes the migration of white blood cells to the vascular wall [14] and triggers pyroptosis in ECs, releasing large amounts of IL-1. These are the initiating factors of AS [15]. IQGAP1 may play an important regulatory role in AS; however, the relationship between IQGAP1 and AS has not yet been studied.

Yes-associated protein (YAP) is a 65-KDa protein in the Hippo signaling pathway that participates in cell proliferation, apoptosis, and other processes [16]. Accumulating evidence suggests that YAP may be a novel target in ASCVD [17]. In *Apoe*^-/-^ mice, EC YAP overexpression promotes AS, whereas YAP knockdown inhibits [18,19]. YAP and TAZ promote EC inflammation and AS by enhancing the JNK signaling pathway in disturbed flow. In contrast, EC inflammation and AS are relieved under unidirectional shear stress by deactivating YAP and TAZ in ECs [18]. YAP is a double-edged sword that regulates cell proliferation and apoptosis [17]; YAP enhances p73-dependent apoptosis but inhibits apoptosis and enhances cell proliferation. However, no studies have examined whether IQGAP1 regulates YAP and apoptosis in ECs, which are critical for AS development.

In the present study, we examined the biological effects of IQGAP1 on AS progression and analyzed its mechanism. We found that IQGAP1 was upregulated in AS tissues. Palmitic acid (PA), one of the most prevalent saturated fatty acids in the modern diet, was used to induce endothelial cell apoptosis. IQGAP1 knockdown reduced EC apoptosis, promoted cell proliferation, and increased YAP expression. In addition, IQGAP1 overexpression resulted in EC apoptosis, reduced cell proliferation and the expression of YAP protein, and increased the phosphorylation of YAP protein.

## 2. Materials and methods

### 2.1 Murine AS model

A total of 16 male C57BL/6 *Apoe*^-/-^ mice were procured from Anhui Medical University. The mice weighed 24 grams and were precisely eight weeks old. After acclimating to their environment for one week, the mice were randomly separated into two cohorts: the high-fat diet cohort (HFD, high fat diet, n = 8) and the standard diet cohort (N, n = 8). The murine AS model was established by feeding mice the HFD for 12 weeks. The HFD comprised 21% milk fat and 0.15% cholesterol (MD12015; Medicience, Yangzhou, China). All animal experiments were conducted in accordance with the guidelines stipulated by the National Institutes of Health Guide for the Care and Use of Laboratory Animals and approved by the Animal Ethics Committee of Anhui Medical University (No. LLSC20221093). To minimize distress, euthanasia was performed under strict ethical guidelines: Anesthesia and Euthanasia: Mice were first anesthetized via inhalation of 3% isoflurane in an induction chamber to reduce pre-euthanasia stress. A lethal dose of pentobarbital sodium (300 mg/kg) was administered intraperitoneally. Death was confirmed by the absence of corneal reflexes, dilated pupils, and cessation of cardiac activity for >5 minutes. Pain Alleviation Measures: Cages were equipped with nesting material (cotton pads), shelters (arched tunnels), and chewable toys to promote natural behaviors. Housing conditions were maintained at 22–24°C and 50–60% humidity with a 12-hour light/dark cycle.

### 2.2 IQGAP1 knockdown in AS mice

To evaluate the role of IQGAP1 in AS, 18 C57BL/6 *Apoe*^-/-^ mice were purchased from Anhui Medical University. The adeno-associated virus 9 carrying IQGAP1-siRNA (AAV-siR-IQGAP1) or the control construct (AAV-NC), both from Hanbio Technology (Shanghai, China), was injected into the tail vein at 2x10^11^ μg per mouse. AAV-siRNA is an effective vector that inhibits the expression of targeted genes in vivo [20]. Afterward, the mice were fed HFD for 12 weeks to create the AS model.

### 2.3 Hematoxylin & eosin (H&E) staining

Briefly, the aortic root tissues of mice were fixed in 4% paraformaldehyde for 24 h. The samples were embedded in paraffin and cut into 3-μm sections. Subsequently, these sections were stained using a hematoxylin solution (C0105S-1; Biyotime, Shanghai, China) for 2 min, followed by staining with hematoxylin for 1 min. Results were visualized using the Panoramic Desk Scanner (3DHISTECH Ltd., Budapest, Hungary).

### 2.4 Oil Red O staining

To evaluate AS, 10-mm frozen aortic sections were immersed in 60% isopropanol and stained with Oil Red O solution at room temperature for 1 h. Sections were visualized using a Panoramic Desk Scanner (3DHISTECH, Ltd.). In addition, the entire aorta was preserved in 4% paraformaldehyde, and the connective tissue surrounding the blood vessels was meticulously removed. The aorta was cut longitudinally and stained with Oil Red O to observe the AS lesions in the entirety of the mouse’s aorta.

### 2.5 TUNEL assay

Cell death was investigated in the mouse aorta using the terminal deoxynucleotidyl transferase (TdT)-mediated dUTP nick end labeling (TUNEL) assay (#C1086; Biyotime, Shanghai, China). Mouse aortic sections were incubated with TdT enzyme for 60 min, followed by treatment with a marked solution at 37°C in conjunction with streptavidin fluorescein for 30 min. Subsequently, the cellular nuclei were stained with 4’,6-diamidino-2-phenylindole for 5 min. The resulting data were obtained using a Panoramic Desktop Scanner (3DHISTECH, Ltd.). The number of TUNEL-positive cells were quantified using ImageJ software, and the average was calculated.

### 2.6 Cell culture and grouping

Human umbilical vein endothelial cells (HUVECs; Clonetics BioWhittaker, East Rutherford, NJ) were cultured in Dulbecco’s Modified Eagle Medium (DMEM; Gibco, NY, USA) supplemented with 10% fetal bovine serum at 37°C in 5% CO_2_. Subsequently, the cultured HUVECs were partitioned into groups, as follows: ***Untreated***, normal control without any treated; ***si-NC***, transfected with negative control siRNA (Jima); ***Si-IQGAP1,*** transfected with small interfering RNA of IQGAP1; ***PA***, 400 μg/ml PA (HY-N0830; Med Chem Express) and negative control siRNA, ***PA + Si-IQGAP1***, 400 μg/ml PA and transfected with small interfering RNA of IQGAP1; ***pcDNA-NC***, transfected with pcDNA3.1-3xFlag-negative control; ***pcDNA-IQGAP1***, transfected with pcDNA3.1-3xFlag-IQGAP1 plasmid; ***Si-IQGAP1 + Si-NC***, transfected with small interfering RNA of IQGAP1 and negative control siRNA of YAP; and ***Si-IQGAP1 + Si-YAP***, transfected with small interfering RNA of IQGAP1 and small interfering RNA of YAP.

### 2.7 siRNA and plasmid vector transfection

To transfect siRNA and plasmid vectors into HUVECs, six-well plates containing 3 × 10^5^ cells per well were cultured for 12–24 h. Subsequently, transfection was performed using jetPRIME (Polyplus-transfection S. A., Illkirch, France) until 60–70% confluence was achieved. The total protein was extracted from cells 24–72 h after transfection, and the transfection efficiency was assessed by western blot analysis.

### 2.8 Flow cytometry

The rate of HUVEC apoptosis was evaluated using the Annexin V-FITC/propidium iodide (PI) apoptosis detection kit (Beyotime, China). HUVECs and the culture medium were obtained from 6-well plates after digestion with trypsin without EDTA. The cells were rinsed twice with PBS, and 1 × 10^5^ cells were collected. Subsequently, 195 μl of Annexin V-FITC binding solution was gently added to suspend the cells, followed by 5 μl of Annexin V-FITC and 10 μl of PI staining solution. The cells were incubated in the dark for 20 min. Detection was carried out using a Beckman CytoFlex flow cytometer (Beckman Coulter Inc.), and data were analyzed using FlowJo software.

### 2.9 Western blot analysis

Proteins were separated using a seven-step process. Then, 5–12% sodium dodecyl sulfate-polyacrylamide gel electrophoresis was performed, and the proteins were subsequently transferred onto a polyvinylidene difluoride (PVDF) membrane (IPVH00010; Merck Millipore Ltd., Darmstadt, Germany). Membranes were blocked using QuickBlock™ Blocking Buffer (Beyotime, Shanghai, China) for 20 min at room temperature and incubated with specific primary antibodies for 16 h at 4°C. The PVDF membranes were then incubated with horseradish peroxidase (HRP)-conjugated anti-rabbit IgG secondary antibodies at room temperature for 90 min. The immune complex was evaluated using an enhanced chemiluminescence system, and the band intensity was analyzed using ImageJ software.

### 2.10 Statistical analysis

The data were expressed as means ± standard deviations and analyzed using the statistical software GraphPad Prism 9.0. To compare the means between two groups, the Student’s t-test was used. Furthermore, to evaluate significant differences among multiple groups, a one-way analysis of variance (ANOVA) was performed, followed by the Student–Newman–Keuls method and Dunnett’s test. p-values less than 0.05 indicated statistical significance.

## 3. Results

### 3.1. Abnormal expression of IQGAP1, cleaved caspase-3, BCL-2, and BAX in the aortic wall of HFD mice and AS plaque

**Fig 1:**
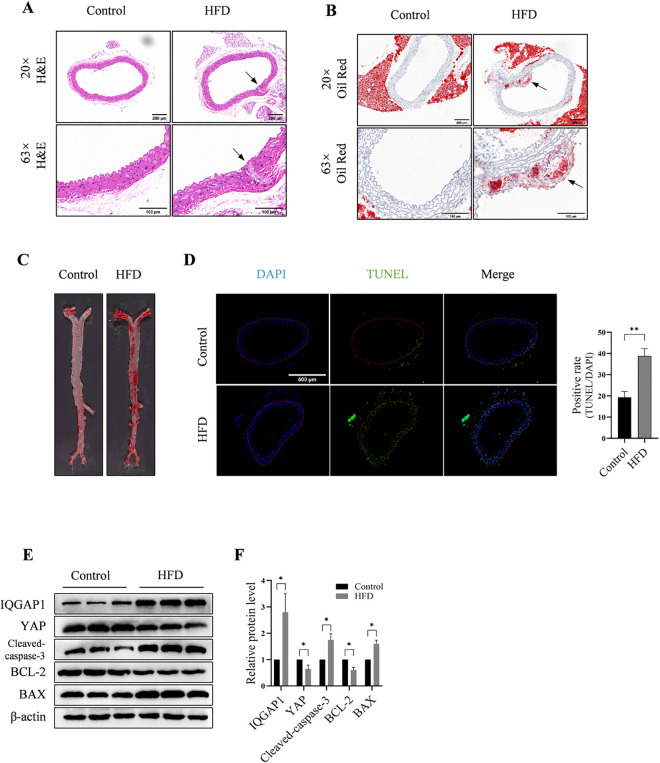
Abnormal expression of IQGAP1, cleaved caspase-3, BCL‐2, and BAX in the aortic wall of HFD mice and AS plaque. (A,B,C) H&E and Oil Red O staining shows the occurrence of AS in the HFD group following 12 weeks of dietary intake. (D) The TUNEL assay demonstrates significantly increased numbers of apoptotic cells in the aortas of HFD mice. (E, F) Western blot and densitometric analysis of each protein relative to that of β‐actin reveals increased expression of IQGAP1 and pro-apoptotic proteins (cleaved caspase-3 and BAX) and decreased YAP and anti-apoptotic protein (BCL-2) expression in the aortic walls of the HFD group. **P < 0.01, ***P < 0.001.

Histological examination of transverse aortic sections that were subjected to H&E (Fig 1A) and Oil Red O staining (Fig 1B) revealed the occurrence of AS in the HFD group after 12 weeks of dietary intake. Oil Red O staining of the entire murine aorta revealed a larger plaque area in the HFD group than in the standard diet group (Fig 1C). The TUNEL assay revealed a significant increase in the number of apoptotic cells in the HFD group (Fig 1D). To detect the expression of IQGAP1 and apoptosis-related proteins in mice with AS, western blot analysis was performed. The expression levels of IQGAP1 and apoptosis proteins were significantly increased in the aortic walls of HFD-fed mice, while the expression levels of YAP and anti-apoptotic protein BCL-2 were decreased. (Figs 1E, 1F). However, whether the elevation of IQGAP1 occurred concomitantly with the development of AS or whether increased IQGAP1 levels resulted in AS was unclear. Therefore, further in vitro and in vivo experiments were performed.

### 3.2. Effect of IQGAP1 on HUVEC apoptosis and proliferation

EC apoptosis is closely associated with AS [21]. PA induces EC damage and apoptosis. Therefore, we used PA as an apoptotic inducer to investigate the effects of IQGAP1 knockdown on PA-induced EC apoptosis. This study investigated the role of IQGAP1 in the apoptotic and atherogenic processes in HUVECs.

**Fig 2:**
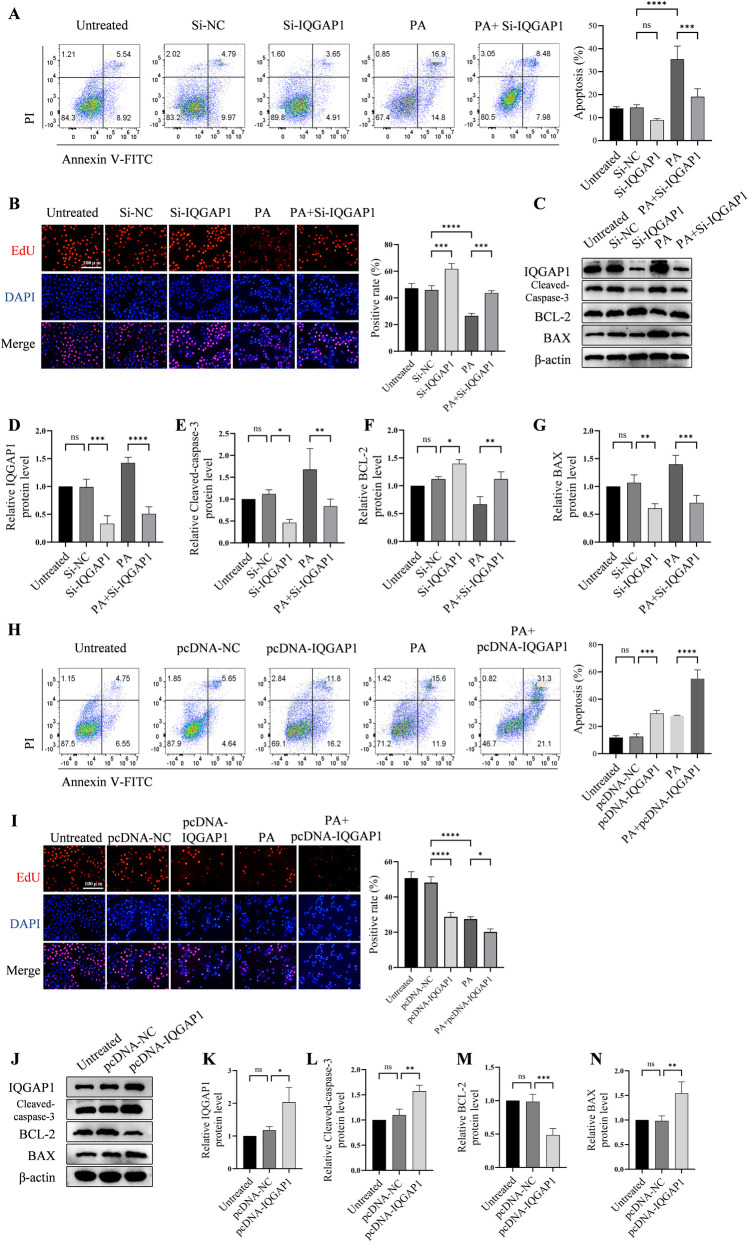
IQGAP1 is a protein that plays a critical role in regulating the level of apoptosis in endothelial cells. (A) The Annexin V–FITC/propidium iodide (PI) assay results indicate that Si-IQGAP1 can slightly decrease the apoptosis rate of normal cells, whereas knocking down IQGAP1 in PA-induced cells (PA + Si-IQGAP1) can significantly reduce the apoptosis rate. (B) The EdU assay reveals that Si-IQGAP1 can slightly increase EC proliferation. PA significantly inhibits HUVEC proliferation, while knocking down IQGAP1 can reverse the PA-induced proliferation stagnation. (C,D,E,F,G) Western blot and densitometric analysis of each protein relative to β-actin demonstrates that Si-IQGAP1 can partially decrease cleaved caspase-3 and BAX expression while enhancing BCL-2 expression. PA can significantly induce the upregulation of apoptotic proteins, but knocking down IQGAP1 can prevent this change, thereby protecting HUVECs from apoptosis. (H) Overexpression of IQGAP1 (pcDNA-IQGAP1) in normal cells can induce apoptosis and a higher apoptosis rate while treated with PA. (I) After transfection with pcDNA-IQGAP1, cell proliferation decreased significantly. (J,K,L,M,N) HUVECs treated with pcDNA-IQGAP1 shows an upregulation of cleaved caspase-3 and BAX expression as well as downregulation of BCL-2 expression. HUVECs, human umbilical vein endothelial cells; EC, endothelial cells; PA, palmitic acid; IQGAP1, IQ motif containing GTPase activating protein 1; Bcl‐2, B‐cell lymphoma 2; Bax, Bcl‐2 associated X; ns P > 0.05, * P < 0.05, ** P < 0.01, *** P < 0.001.

Annexin V–FITC/ PI staining revealed that the apoptotic rate in the Si-IQGAP1 group was approximately 5% lower than that in the normal group, but the differences were not significant. This may be attributed to the fact that the apoptotic rate of normal cells, without PA induction, is relatively low. After PA induction, the apoptotic rate of the cells increased from 13% to 32%. The knockdown of IQGAP1 and induction of apoptosis with PA decreased the apoptotic rate from 32% to 18% (Fig 2A). Changes in apoptosis levels are often accompanied by changes in cell proliferation; therefore, we also measured cell proliferation levels. The EdU assay revealed that cell proliferation was increased in the Si-IQGAP1 group without or with PA treatment compared to that in the Si-NC and PA groups, respectively (Fig 2B). Western blotting was performed to detect the expression of apoptosis-related proteins. Within the PA cohort, the upregulation of the pro-apoptotic protein cleaved caspase-3 and BAX occurred concomitantly with the downregulation of the anti-apoptotic protein BCL-2. However, after IQGAP1 knockdown, the changes in protein expression were reversed (Figs 2C–2G).

The cell apoptosis rate was significantly increased in pcDNA-IQGAP1 group (Fig 2H). PA can also induce apoptosis, just as overexpression of IQGAP1 in cells. In the presence of PA, overexpression of IQGAP1 will further aggravate apoptosis (Fig 2H). The EdU-positive ratio was lower in the pcDNA-IQGAP1 group than in the pcDNA-NC group. The use of PA will lead to more decrease in cell proliferation. (Fig 2I). Treated with pcDNA-IQGAP1 in normal cells can lead to an upregulation of cleaved caspase-3 and BAX expression as well as downregulation of BCL-2 expression (Figs 2J–2N).

In general, these results indicated that the reduction in IQGAP1 expression may hinder programmed cell death or apoptosis in HUVECs activated by PA. In contrast, enhanced IQGAP1 expression may trigger HUVEC apoptosis.

### 3.3. Impact of IQGAP1 on YAP

**Fig 3:**
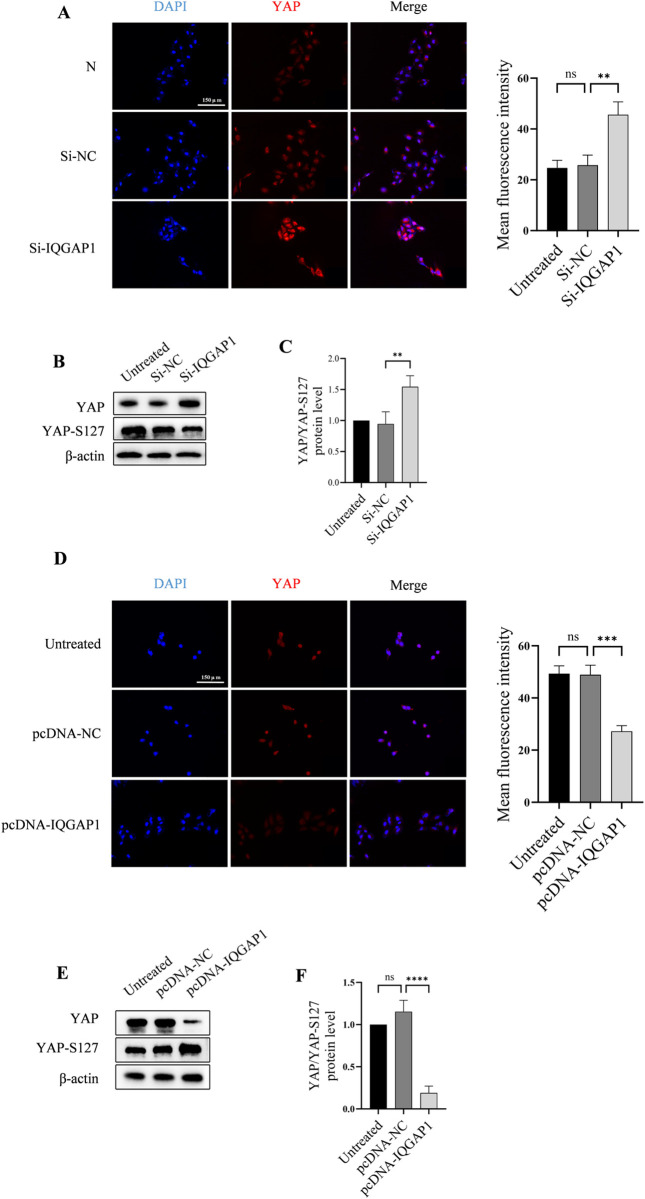
IQGAP1 alters the expression of YAP. (A) Semi-quantitative immunofluorescence analysis indicates that YAP expression increases upon IQGAP1 knockdown. (B) Western blot analysis shows that Si-IQGAP1 increased YAP expression and inhibits the phosphorylation of the S127 site of YAP. (C) Active intracellular YAP expression were decreased in Si-IQGAP1 group. (D) Semi-quantitative immunofluorescence analysis indicates that overexpression of IQGAP1 can decrease the expression of YAP. (E, F) Overexpression of IQGAP1 can reduce the proportion of YAP/ YAP-S127 and increase the phosphorylation of YAP at S127 site. ns P > 0.05, **P < 0.01, ***P < 0.001.

The downstream effector protein YAP is a critical regulator of cell proliferation and apoptosis in the Hippo signaling pathway. This study aimed to explore the possible correlation between alterations in IQGAP1 and YAP protein expression. Our immunofluorescence data demonstrate that YAP is localized in both the nucleus and cytoplasm. Semi-quantitative immunofluorescence analysis revealed significant upregulation of YAP expression in Si-IQGAP1 group (Fig 3A). Western blot and semiquantitative fluorescence analysis further demonstrated that IQGAP1 knockdown caused an increase in YAP expression but a slight decrease in YAP-S127 expression (Fig 3B). YAP/YAP-S127 was used as a relative measure of active intracellular YAP expression and the results showed active intracellular YAP expression was increased after treated with Si-IQGAP1(Fig 3C). In contrast, the overexpression of IQGAP1 (pcDNA-IQGAP1) in HUVECs resulted in decreased YAP protein expression by Semi-quantitative immunofluorescence analysis (Fig 3D). YAP protein and active intracellular YAP expression were decreased,but YAP-S127 was increased in pcDNA-IQGAP1 group (Fig 3E, 3F).

These findings suggest that IQGAP1 promoted the phosphorylation and degradation of YAP and inhibited the expression of YAP in cells. As a transcription cofactor, YAP can translocate into the nucleus and regulate gene expression, thereby affecting various cellular processes such as growth, development, proliferation, and apoptosis [16]. Our results indicate that IQGAP1 is involved in the expression and stability of YAP.

### 3.4. YAP knockdown reversed the effects of HUVEC apoptosis and proliferation caused by IQGAP1 knockdown

**Fig 4:**
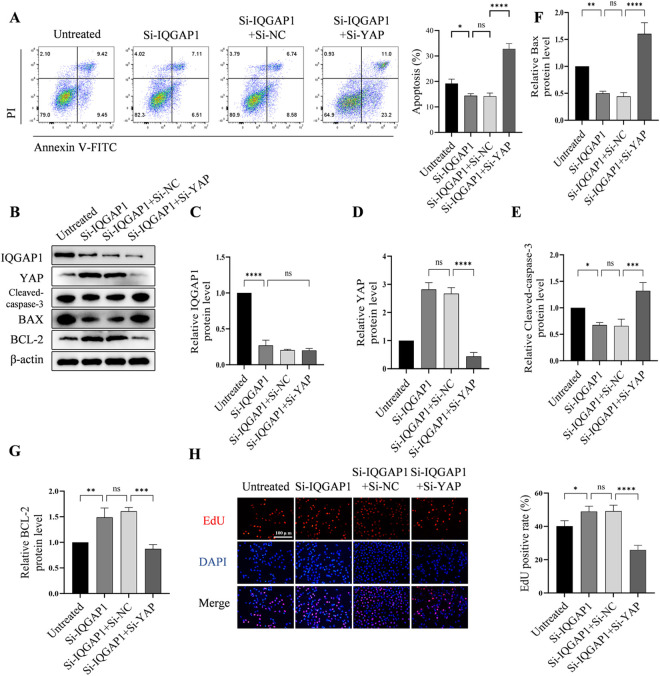
Si-YAP reverses the apoptosis induced by the knockdown of IQGAP1 and decreased cell proliferation. (A) Flow cytometry results indicate that inhibiting YAP expression using Si-YAP increased the HUVEC apoptosis. (B,C,D,E,F,G)) Western blot analysis reveals that when both Si-IQGAP1 and Si-YAP are co-transfected, Si-YAP increased the expression of cleaved caspase-3 and BAX as well as the decrease in BCL-2. (H) The EdU analysis demonstrates that Si-YAP can decline the cell proliferation. ns P > 0.05, **P < 0.01, ***P < 0.001.

To determine whether YAP was involved in the regulation of HUVEC proliferation and apoptosis by IQGAP1, we performed rescue experiments. The small interfering RNA of IQGAP1 (Si-IQGAP1) and small interfering RNA of YAP (Si-YAP) were co-transfected into HUVECs (Si-IQGAP1 + Si-YAP group). Annexin V–FITC/ PI staining revealed that Si-IQGAP1 decreased the apoptosis of HUVECs, but co-transfection with Si-IQGAP1 and Si-YAP increased the apoptosis rate (Fig 4A). Western blot analysis revealed decreased expression of IQGAP1, YAP and BCL-2 and increased expression of Cleaved caspase-3 and BAX in Si-IQGAP1 + Si-YAP group (Figs 4B–4G). EdU staining results indicated that Si-YAP reversed the increased HUVEC proliferation rate caused by Si-IQGAP1 (Fig 4H).

### 3.5. IQGAP1 knockdown limits atherosclerotic lesion formation

**Fig 5:**
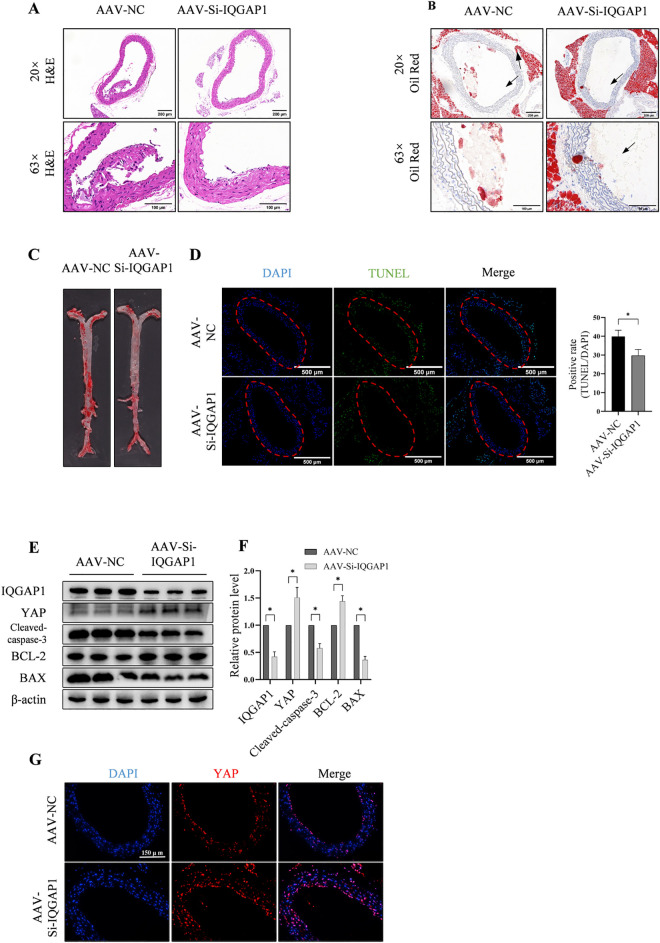
Inhibiting IQGAP1 expression restricts atherosclerotic plaque development. (A) H&E and (B, C) Oil Red O staining shows AS in the AAV-NC and AAV-Si-IQGAP1 groups following 12 weeks of HFD. However, the atherosclerotic plaque size and area in AAV-Si-IQGAP1 mice are smaller than those in the AAV-NC group. (D) The TUNEL assay demonstrates that the number of apoptotic cells are significantly decreased in the AAV-Si-IQGAP1 group. (E,F) Western blot and densitometric analyses of each protein relative to β‐actin reveal decreased expression of IQGAP1, and pro-apoptotic proteins (cleaved caspase-3 and BAX), while YAP and the anti-apoptotic protein BCL-2 are increased in the aortic wall of the AAV-Si-IQGAP1 groups. (G) IQGAP1 knockdown also leads to an increase of YAP in mouse aortic. HFD, high-fat diet; AS, atherosclerosis; IQGAP1, IQ motif containing GTPase activating protein 1; Bcl‐2, B‐cell lymphoma 2; Bax, Bcl‐2 associated X. **P < 0.01, ***P < 0.001.

Through H&E and Oil Red O staining of aortic root sections, we found that the level of AS in *Apoe*^-/-^ mice treated with AAV-Si-IQGAP1 was lower than that in *Apoe*^-/-^ mice treated with AAV-NC. (Figs 5A–5C) TUNEL staining of aortic root sections revealed that compared with AAV-NC mice fed an HFD for 12 weeks, mice treated with AAV-Si-IQGAP1 had fewer apoptotic cells in the aortic root (Fig 5D). The western blotting results demonstrated decreased IQGAP1, Cleaved caspase-3, BAX and increased YAP, BCL-2 expression in AAV-Si-IQGAP1 group (Figs 5E,5F). Immuno-histochemical staining demonstrated more YAP in the aortic roots of AAV-Si-IQGAP1 group, which validated that IQGAP1 knockdown could lead to an increase of YAP in atherosclerotic plaques of arteries (Fig 5G).

## 4. Discussion

AS is the primary and complex pathological basis of ASCVD; however, a complete understanding of the underlying molecular mechanisms remains incomplete. EC apoptosis is regulated by multiple stimuli, including oxidized low-density lipoproteins (ox-LDL), PA, and lipopolysaccharides (LPS). Signaling pathways and immune responses play crucial roles in AS initiation and progression. Thus, interventions that target these etiological factors or signaling pathways have the potential to slow or even stop AS progression.

This study confirms the direct impact of lipid deposition on EC apoptosis, both in vitro and in vivo. Our results also show that PA increases IQGAP1 expression in ECs. IQGAP1 knockdown increases YAP protein levels and inhibits PA-induced apoptosis in HUVECs. Conversely, IQGAP1 overexpression can reduce YAP, and lead to EC apoptosis. These results indicate that IQGAP1 can control EC apoptosis and participate in the AS regulatory process by regulating the YAP.

IQGAP1, a large 190 kDa protein (1,657 amino acids), is widely expressed in cells. The protein has five major structural domains that can bind to other proteins: a calponin homology domain (CHD), a polyproline protein-protein binding domain (WW), a domain containing four IQ motifs (IQ), a Ras GAP-related domain (GRD), and a Ras GAP C-terminal domain (RGCT) [6]. These binding domains mediate the interaction of IQGAP1 with hundreds of proteins, thereby participating in various biological activities, such as cell cytoskeletal dynamics, cell-cell adhesion, cell migration/invasion, apoptosis, and cell proliferation [6,22]. Previous studies have primarily focused on the role of IQGAP1 in tumorigenesis. Liu et al. [9] showed that IQGAP1 interacts with the ERK1/2 signaling pathway, promotes ERK1/2 phosphorylation, and contributes to Ang II-induced apoptosis. Sbroggiò et al. [23] suggested that IQGAP1 knockout in mice leads to increased apoptosis in response to pressure overload in the heart, which is mediated by the Raf-MEK1/2-ERK1/2 signaling pathway. The regulation of IQGAP1 in cells and signaling pathways may be heterogeneous in different cell lines or tissues. Our research shows that IQGAP1 expression is upregulated in AS and that apoptosis is also increased. Subsequent rescue experiments indicate that as a member of this signaling pathway, IQGAP1 promotes EC apoptosis. We conducted preliminary investigations into the mechanism by which IQGAP1 regulates EC apoptosis.

Aberrations in the Hippo pathway and YAP/TAZ hyperactivation have been observed in many human malignancies. YAP is retained in the cytoplasm by binding to 14-3-3 [24] or is degraded through ubiquitination [25], preventing its participation in gene transcription when phosphorylated at S127. In contrast, dephosphorylated YAP/TAZ can translocate to the nucleus and promote target gene transcription through interactions with transcription factors such as TEAD1–4 [26]. Recent studies have shown that the Hippo signaling pathway is an important target in cardiovascular diseases [17]. According to Wang et al., the nuclear localization of YAP/TAZ notably increases, with a concomitant increase in the expression of pro-inflammatory target genes, within the endothelium in regions afflicted with AS lesions [27]. YAP is a gene that regulates cellular processes such as proliferation and apoptosis and exhibits a dichotomous nature. On the one hand, YAP promotes downstream target genes such as β-catenin [28], connective tissue growth factor (CTGF), and angiogenic inducer 61 (Cyr61) [29].

This study has some limitations. While this study primarily focuses on the impact of IQGAP1 protein on atherogenesis and provides preliminary investigation into IQGAP1’s regulatory effect on YAP, we acknowledge that the in vivo validation of YAP protein and HIPPO signaling pathway involvement in atherosclerotic progression remains unexplored. The current experimental design did not perform systematic verification of whether YAP acts as the downstream executor of IQGAP1 in modulating vascular inflammation or lipid deposition. Future studies employing IQGAP1/YAP double knockout murine models should be conducted to dissect their synergistic mechanisms in atherosclerosis development. Such investigations would help clarify whether these two molecules function through parallel pathways or constitute sequential components within the same pathological cascade, thereby providing deeper insights into the molecular orchestration of vascular remodeling processes.

In summary, our research established that IQGAP1 is an important factor that leads to apoptosis of endothelial cells and atherosclerosis in the aorta of mice. IQGAP1 knockdown results in increased YAP expression, thereby exerting a protective effect against EC apoptosis and AS. Conversely, IQGAP1 upregulation decreased YAP expression.

## Supporting information

S1 Data This contains the complete raw dataset supporting all study results, along with associated statistical analyses as described in Methods Section. (XLSX)

S1 File This contains the original Western Blot images for Figures, as previously submitted to the editorial office. (PDF)
