# Supplementary material for: IQGAP1 participates in endothelial cell apoptosis and regulates atherosclerosis by targeting YAP
Source: PLoS One. 2025 Jul 14;20(7):e0328345. doi: 10.1371/journal.pone.0328345 (PMC12258547; doi:10.1371/journal.pone.0328345)
Supplement: S1 File — (PDF) [file pone.0328345.s002.pdf]

150kd

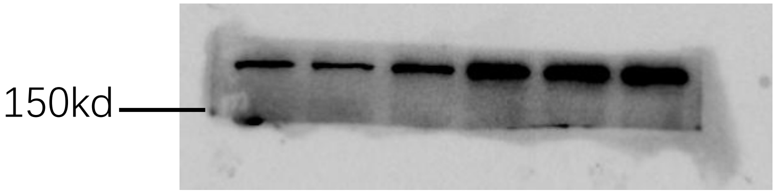

IQGAP1 for Fig 1E

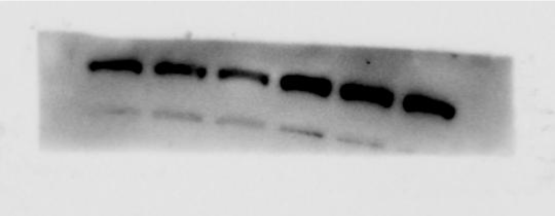

Caspase-3 for Fig 1E

35kd
25kd

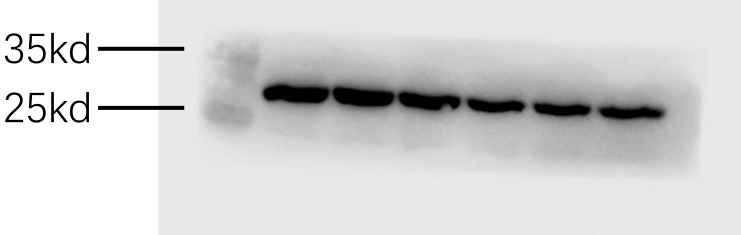

BCL-2 for Fig 1E

15kd

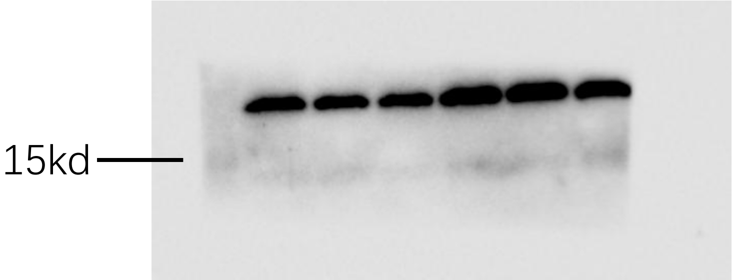

BAX for Fig 1E

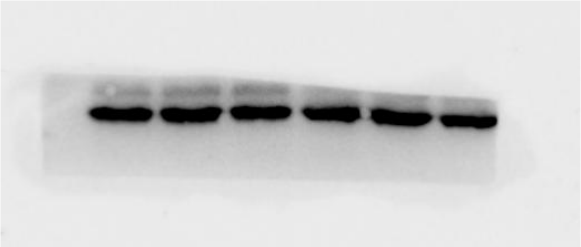

β-actin for Fig 1E

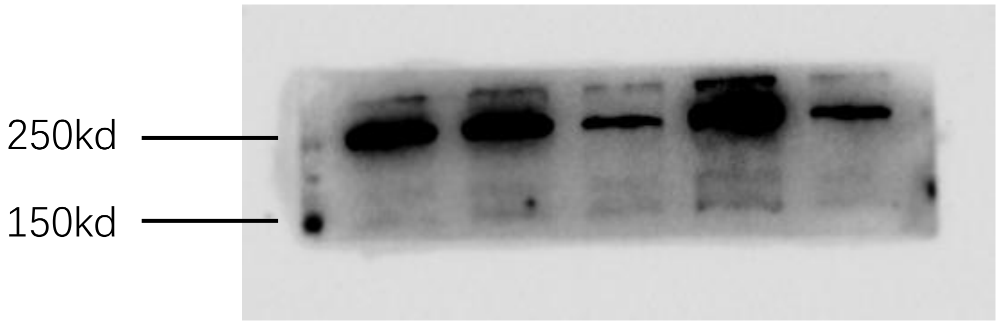

IQGAP1 for Fig 2C

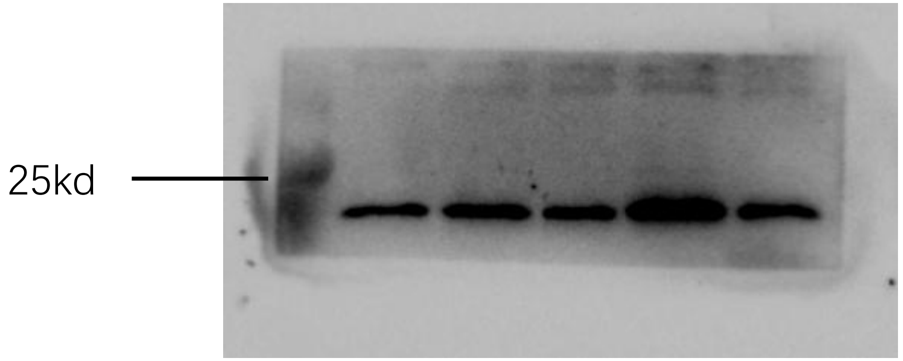

BAX for Fig 2C

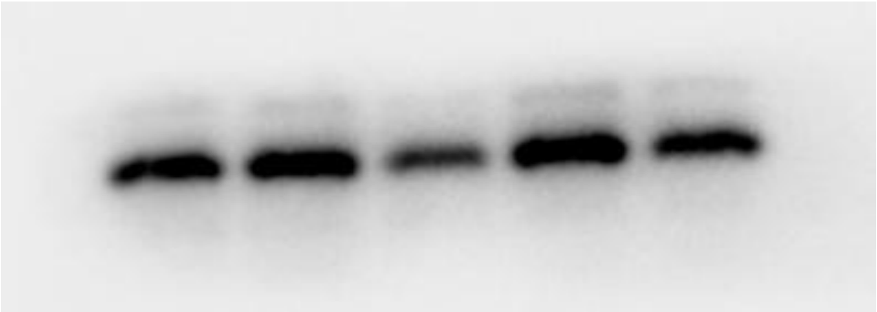

Caspase-3 for Fig 2C

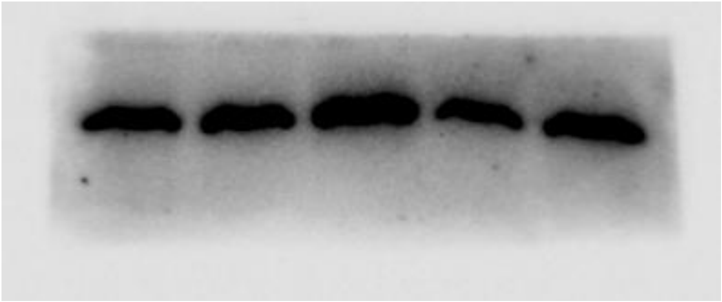

BCL-2 for Fig 2C

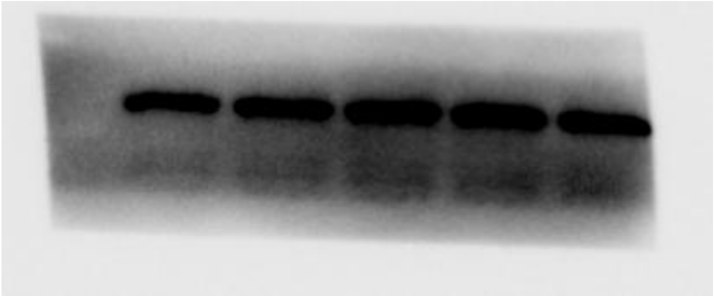

β-actin for Fig 2C

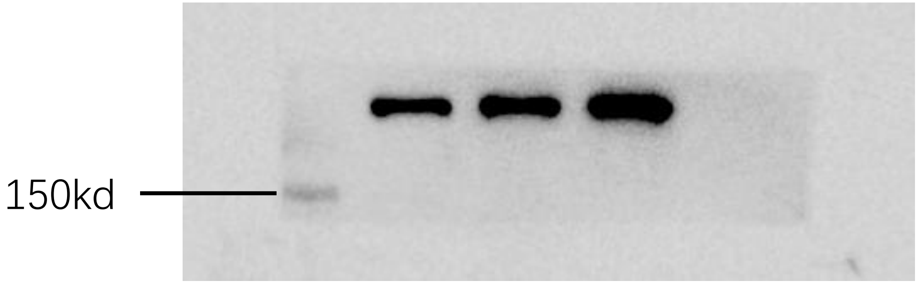

IQGAP1 for Fig 2J

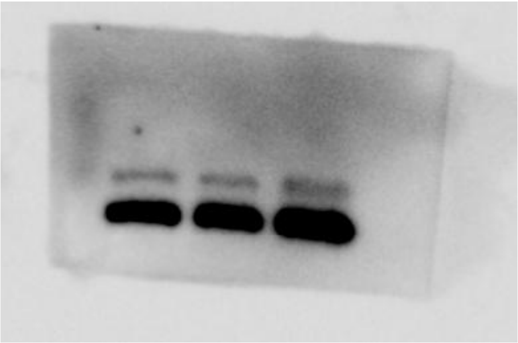

Caspase-3 for Fig 2J

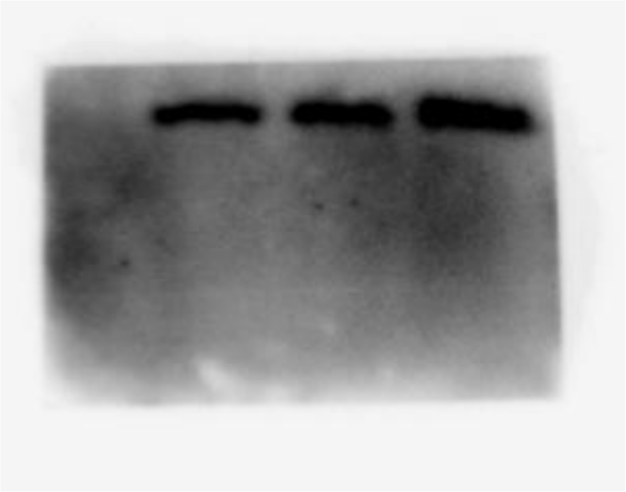

BAX for Fig 2J

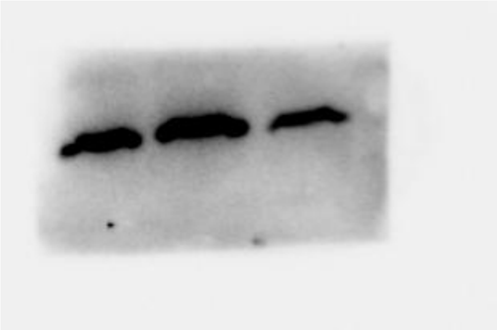

BCL-2 for Fig 2J

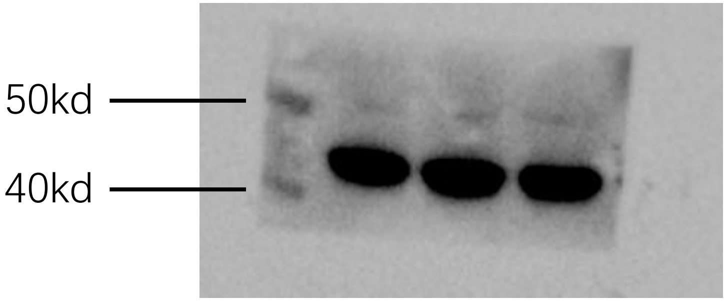

β-actin for Fig 2J

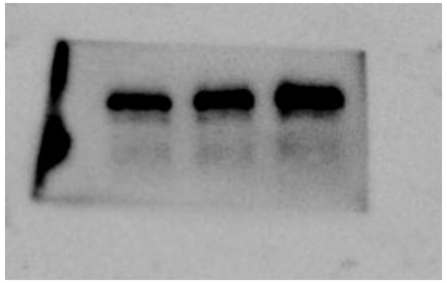

YAP for Fig 3E

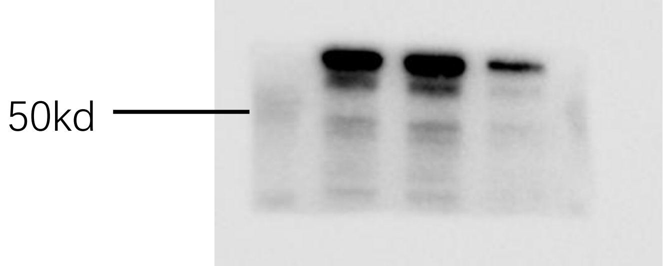

YAP-S127 for Fig 3E

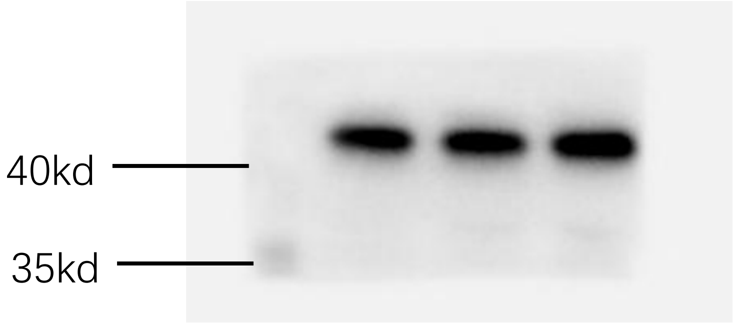

β-actin for Fig 3E

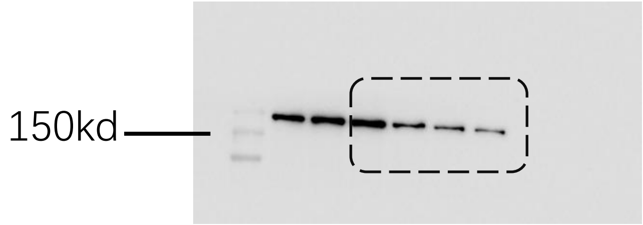

YAP for Fig 4B

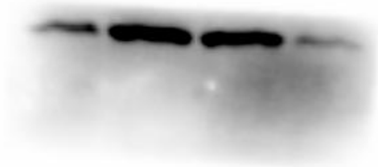

YAP for Fig 4B

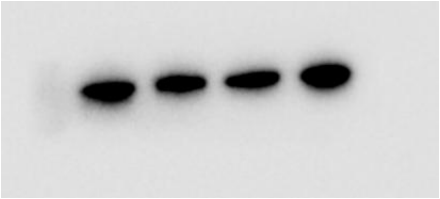

Caspase-3 for Fig 4B

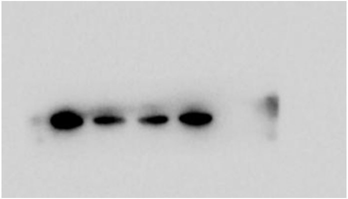

BAX for Fig 4B

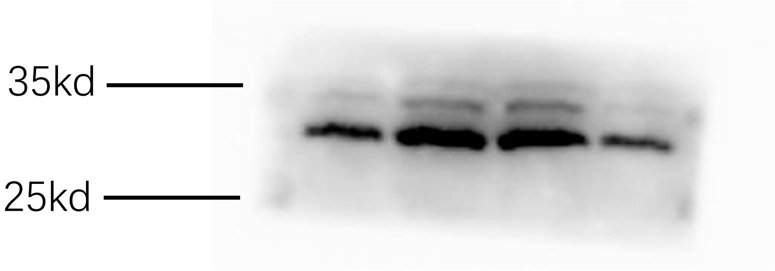

BCL-2 for Fig 5A

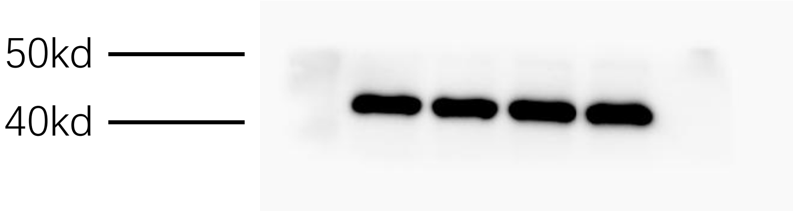

β-actin for Fig 5A

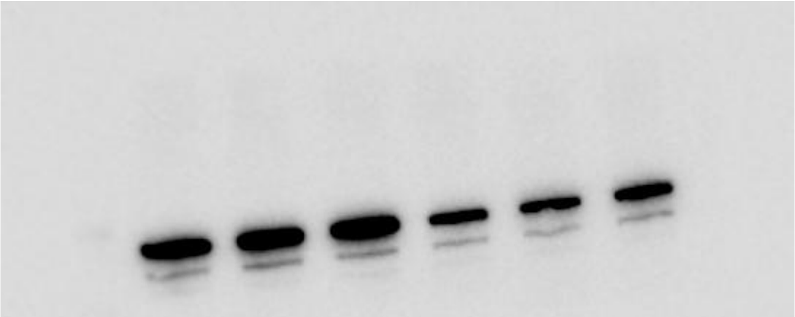

IQGAP1 for Fig 5E

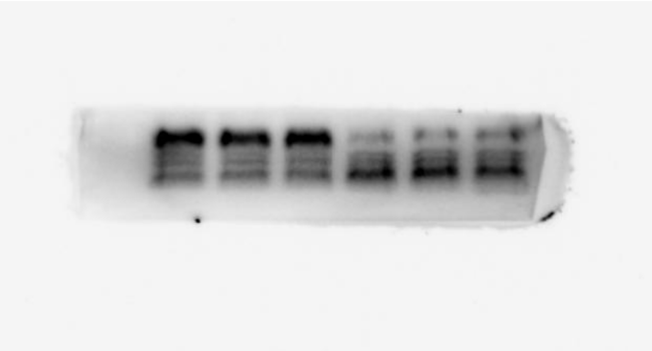

YAP for Fig 5E

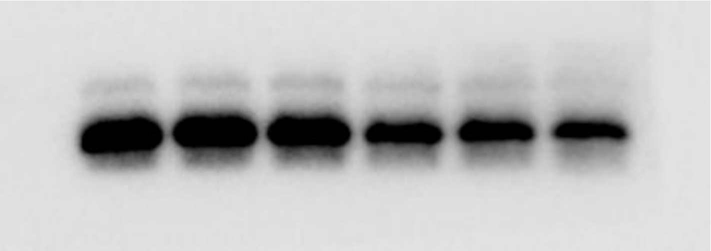

Caspase-3 for Fig 5E

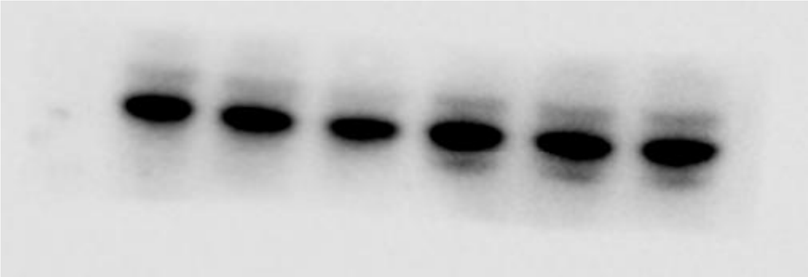

BCL-2 for Fig 6E

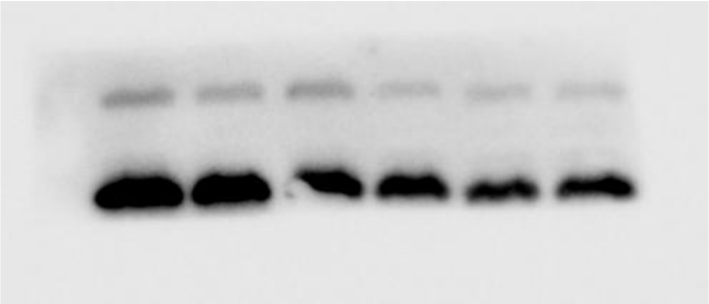

BAX for Fig 6E

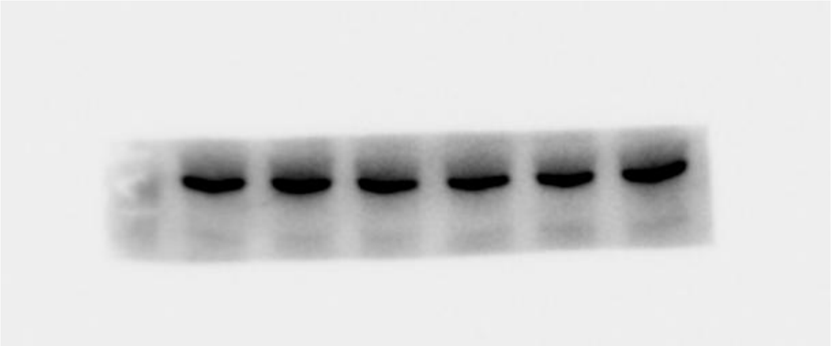

B-actin for Fig 6E
